# Health Assessment of Trace Metal Concentrations in Organic Fertilizer in Northern China

**DOI:** 10.3390/ijerph16061031

**Published:** 2019-03-21

**Authors:** Qiong Gong, Peizhen Chen, Rongguang Shi, Yi Gao, Shun-An Zheng, Yan Xu, Chaofeng Shao, Xiangqun Zheng

**Affiliations:** 1 Agro-Environmental Protection Institute, Ministry of Agriculture, Tianjin 300191, China; gongqiong9104@163.com (Q.G.); winsomesky@163.com (R.S.); 17775399050@163.com (Y.G.); xuyan_wa@163.com (Y.X.); 2 Rural Energy and Environment Agency, Ministry of Agriculture and Rural Affairs, Beijing 100125, China; zhengshunan1234@163.com; 3 College of Environmental Science and Engineering, Nankai University, Tianjin300350, China; shaocf@nankai.edu.cn

**Keywords:** organic fertilizer, trace metal, soil accumulation risk, human exposure, human health risk

## Abstract

The application of organic fertilizer could be accompanied by potential hazards to soil and humans caused by trace metals. A wide survey of organic fertilizers was carried out in northern China. A total of 117 organic fertilizer samples were collected to analyze the concentrations of seven trace metals. Simulation models were used to estimate the trace metal accumulation risk in soil and non-carcinogenic and carcinogenic risks to the human body. The concentrations of trace metals varied widely (Cr: 2.74–151.15; Ni: 2.94–49.35; Cu: 0.76–378.32; Zn: 0.50–1748.01; As: 1.54–23.96; Cd: 2.74–151.15; and Pb: 1.60–151.09 mg·kg^−1^). Chinese organic fertilizer standard limits were exceeded by0.85% for Cr, 5.98% for As, 1.71% for Cd, and 4.27% for Pb. Monte Carlo simulations showed that repeated application of organic fertilizer likely significantly increased the concentrations of Zn, Cd, and As in soil compared with the soil background levels according to the Soil Environmental Quality Standards of China. As and Cr pose high risks to human health, especially as carcinogenic risk factors with a skin exposure pathway. Reducing the content of Cr, Cu, Zn, As, and Cd in organic fertilizer would be of great significance for minimizing the damage caused by trace metals.

## 1. Introduction

The consumption of fertilizers in China is widespread. According to the China Statistical Yearbook (1953–2017), the annual use of chemical fertilizers in cultivated land increased from 78,000 tons in 1952 to 59.841 million tons in 2016, thus improving food production [1]. However, at the same time, increased fertilizer use has given rise to problems such as greenhouse gas emissions and compaction and acidification of soil [2,3,4]. To this end, in recent years the Ministry of Agriculture and Rural Affairs of the People’s Republic of China has issued a series of action plans, such as the “Action Plan for Zero Growth of Fertilizer Consumption until 2020” in 2015 (Nong Nong Fa [2015] NO.2) and the “Replacing Chemical Fertilizer with Organic Fertilizer (Fruits, Vegetables, and Tea) Action Plan” in 2017 (Nong Nong Fa [2017] NO.2), to improve organic fertilizer use, reduce unreasonable investment, ensure sufficient supply of grains and other major agricultural products, and promote the sustainable development of agriculture. The use of organic fertilizers is not only a means to effectively reuse agricultural waste resources, but also an agricultural waste management method with cost and environmental benefits. Organic fertilizers are also a critical link in the virtuous cycle of livestock–soil–plant systems [5,6,7]. Agricultural waste, including livestock manure and crop straw, can stabilize nutrients through aerobic fermentation, thus becoming safer and more stable fertilizers (organic fertilizers) [8].

Organic fertilizers are a beneficial source of plant nutrients and organic matter. Farmers often use it in plant production to increase productivity. However, organic fertilizers can also be a potential source of environmental pollution. There is increasing evidence that organic fertilizers can have high concentrations of trace metals such as Cr, Ni, Cu, Zn, As, Cd, and Pb. If organic fertilizers are applied to an agricultural area, some trace metals could be accumulated in agricultural lands, some of which could be transferred to the human body [9,10]. Therefore, investigating the trace metal contents of organic fertilizers is important for preventing the contamination of soil, water, and livestock [11,12]. Some researchers have conducted large-scale research on trace metal and nutrient contents in Chinese organic fertilizers [13,14]. The trace metal contents of these fertilizers exceeded the standard values. For example, the concentrations of Cr, As, Cd, and Pb in 212 samples of Chinese organic fertilizers containing livestock manure exceeded the standard value by 4.2%, 13.7%, 2.4%, and 1.4%, respectively [13]. The presence of trace metals in organic fertilizers has also become an important reason for limiting the use of these fertilizers. It is important to understand the status and extent of soil contamination of trace metals from organic fertilizers to develop sustainable management strategies for agricultural soils. It has been reported that atmospheric deposition and livestock manure were the predominant sources of trace metals entering agricultural soils in China [15], with atmospheric deposition responsible for 43–85% of the total As, Cr, Hg, Ni, and Pb inputs, and livestock manure accounting for approximately 55%, 69%, and 51% of the total Cd, Cu, and Zn inputs, respectively [16]. According to Ding et al., compost and atmospheric deposition were sources of pollution into agricultural land, with compost contributing 12.82–89.06% of trace metals (Zn: 36.03%; Ni: 51.50%; Cu: 42.52%; Zn: 73.94%; As: 12.82%; Cd: 89.06%; and Pb: 64.22%) [17]. It can be seen that organic fertilizer was the major source contributing trace metals to the environment, with manure and compost being the two kinds of organic fertilizer [17,18], even though other activities (such as atmospheric deposition or mining) could also cause soil accumulation [19]. Analysis of the trace metal content of organic fertilizers and estimation of the time (in years) required for trace metal concentration in soil to exceed limit values are beneficial for monitoring the soil environmental quality, ensuring the safety of agricultural products, and protecting the ecosystem.

Trace metals can also cause harm to human health, and occupational exposure increases the risk of this. According to the list of carcinogens published by the World Health Organization’s International Agency for Research on Cancer on 27 October 2017, As, Cd, Cr(VI), and Ni compounds are a class of carcinogens. Inorganic Pb is a Class 2A carcinogen, and Ni, Pb, and Cr(III) are Class 2B carcinogens (http://samr.cfda.gov.cn/WS01/CL1991/215896.html). Occupational exposure increases the risk to human health, and long-term exposure to trace metals increases the risk of non-carcinogenic and carcinogenic cells [20]. There are three main ways in which the human body can be exposed to trace metals in the soil: (1) oral absorption; (2) absorption by dermal contact (skin exposure); and (3) inhalation of floating soil particles (respiratory exposure) [21,22,23]. Studying the carcinogenic and non-carcinogenic risks to human health through these three exposure pathways can not only reveal high-risk exposure pathways and trace metal species, but they can also help farmers to avoid risks in their agricultural businesses [24].

In summary, this study used chemical testing and simulation prediction methods to achieve the following objectives: (1) determination of concentrations and above-standard levels of seven trace metals (Cr, Ni, Cu, Zn, As, Cd, and Pb) in organic fertilizers produced in different regions of northern China; (2) estimation of potential risks of organic fertilizer application to the accumulation of trace metals in the soil; and (3) simulation of potential human health risks following exposure to organic fertilizer containing trace metals.

## 2. Materials and Methods

### 2.1. Sample Collection and Processing

#### 2.1.1. Sample Collection and Pre-Treatment

**Figure 1:**
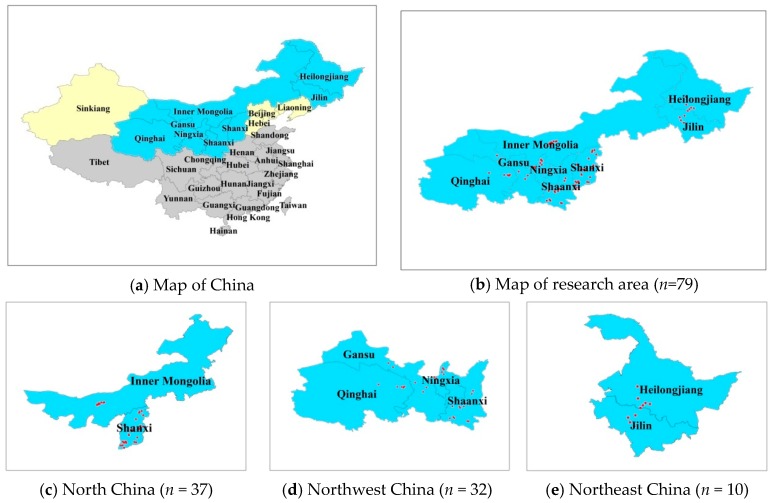
Sketch map of research areas and sampling points in northern China. (**a**) Sketch map of research areas; the blue and yellow areas denote northern China, with the blue indicating the research area of this article. (**b**) Sampling points in northern China (*n* = 79), including Northwest China, North China, and Northeast China. (**c**) Sampling points in Northwest China (*n* = 32), including Shaanxi, Ningxia, Gansu, and Qinghai Provinces. (**d**) Sampling points in North China (*n* = 37), including inner Mongolia and Shanxi Provinces. (**e**) Sampling points in Northeast China (*n* = 10), including Heilongjiang and Jilin Provinces. Note: *n* denotes the number of sampled organic fertilizer enterprises.

Samples were obtained from organic fertilizer factories in eight provinces in northern China from June to August 2017 (Figure 1). These factories collected manure (Table 1) from local livestock farms and produced organic fertilizers by commercial operation. Each organic fertilizer sample consisted of three subsamples. The subsamples were collected from five points from a single composted pile, mixed thoroughly into a 2–3 kg sample, and transported to the laboratory. Samples were dried at 70 °C for 48 h, and ground to pass through a 100-mesh (0.15 mm) sieve prior to analysis. The remaining samples were stored at <−80 °C until analysis.

#### 2.1.2. Sample Preparation and Analysis for the Total Concentration of Trace Metals

All glassware and plastic containers were immersed in 5% (v/v) nitric acid solution for 24 h and then rinsed with ultrapure water and reserved for use. The dried samples were analyzed for trace metal contents in accordance with Wang et al. and Hseu [25,26]. In brief, samples were digested in HNO_3_–HClO_4_ (80/20, v/v) and concentrations of Cr, Ni, Cu, Zn, As, Cd, and Pb were analyzed by inductively-coupled plasma mass spectrometry (7900 ICP-MS, Agilent Technologies, Santa Clara, CA, USA). Method blanks, duplicate samples, and soil standard samples (GBW07456, Geophysical and Geochemical Exploration Institute of the Chinese Academy of Geological Sciences) were added in the analytical process of each batch sample for quality assurance and control.

### 2.2. Risk of Trace Metal Accumulation in Soil from the Applications ofFertilizer

To provide meaningful information about the risk of trace metal accumulation in farmland soil caused by the application of organic fertilizers, Monte Carlo simulations were used to estimate the long-term effects of applications on seven trace metal concentrations in soil [13,17].

#### 2.2.1. Accumulation of Trace Metals in Soil

The accumulation of trace metals in the soil is the total value of soil background and the amount of trace metals in organic fertilizer.

(1) $$Q_{i}=Q_{p,i}+Q_{0,i}$$

where *i* refers to the seven trace metals, Cr, Ni, Cu, Zn, As, Cd, and Pb; $Q_{i}$ is the cumulative amount of contaminant in the soil, in mg·kg^−1^; $Q_{p,i}$ is the input amount of contaminant *i*, in mg·kg^−1^; and $Q_{0,i}$ is the initial content of contaminant *i* in the soil, in mg·kg^−1^, which is the average of the study area (Table 1).

#### 2.2.2. Amount of Trace Metals in Soil from Organic Fertilizer

The accumulation of trace metals in the soil was assessed by the input fluxes of organic fertilizer:

(2) $$Q_{p,i}=\frac{IR_{f}\times \alpha_{i}}{\rho_{b}\times V}$$

where $Q_{p,i}$ is the input of contaminant *i*, in mg·kg^−1^; $IR_{f}$ is the amount of organic fertilizer applied to the soil, in t·kg^−1^·year^−1^ (Table 1), which is the average of the study area; $\alpha_{i}$ is the median content of contaminant in the organic fertilizer, in mg·kg^−1^; $\rho_{b}$ is the soil bulk density, in g·cm^−3^ (Table 2); and *V* is the volume of soil per hectare of the plow layer (20 cm), 2 × 10^3^ m^3^·hm^−2^.

#### 2.2.3. Time Scale (in Years) Needed to Double the Trace Metal Concentrations in Soil from Its Background Levels

Analysis of the trace metal content of organic fertilizers and estimation of the time (in years) required for trace metal concentration in soil to exceed limit values can be calculated using the following equation:

(3) $$T_{i}=\frac{C_{s,i}-Q_{0,i}}{Q_{p,i}}$$

where $T_{i}$ is the time scale (in years) needed to double the trace metal concentrations in soil from its background levels according to the Soil Environmental Quality Standard of China (GB15618-2018); $C_{s,i}$ is the limit value of dry land in contaminant *i* at pH > 7.5, according to the Soil Environmental Quality Agricultural Soil Risk Control Standards (GB15618-2018); and $Q_{s,i}$ of Cr, Ni, Cu, Zn, As, Cd, and Pb are 250, 190, 100, 300, 25, 0.6, and 170 mg·kg^−1^, respectively.

### 2.3. Human Body Exposure Model for Trace Metals

#### 2.3.1. Oral Exposure

The concentration of trace metals entering the body through food can be calculated as follows:

(4) $$Q_{f,i}=\frac{Q_{i}\times IR_{p}\times EF_{a}\times ED_{a}\times \beta_{i}}{BW\times LT}\times 10^{-6}$$

where $Q_{f,i}$ is the concentration of the contaminant *i* entering the human body through the mouth, in mg·kg^−1^·day^−1^; $IR_{p}$ is the adult intake, 100 mg·day^−1^ [33]; $EF_{a}$ is the exposure frequency, 250 day·year^−1^ [33]; $ED_{a}$ is the length of exposure, 25 years [33]; $\beta_{i}$ is the absorption coefficient of contaminant in the stomach, where the gastrointestinal absorption coefficients for Cr, Ni, Cu, Zn, As, Cd, and Pb are 2.5 × 10^−2^, 1.6 × 10^−2^, 1.00, 1.00, 1.00, 5.00 × 10^−2^, and 1.00, respectively [10]; *BW* is the average weight of adults, 56.8 kg [33]; and *LT* is the human exposure time, 26,280 days [33].

#### 2.3.2. Skin Exposure

The concentration of trace metals entering the body through the skin can be calculated as follows:

(5) $$Q_{s,i}=\frac{Q_{i}\times E_{V}\times SA\times \mu \times EF_{a}\times ED_{a}\times \eta \times \gamma_{i}}{BW\times LT}\times 10^{-6}$$

where $Q_{s,i}$ is the concentration of contaminant *i* entering the body through the skin, in mg·kg^−1^·day^−1^; $E_{v}$ is the skin contact frequency, 1 day^−1^ [33]; SA is the skin surface area, 1.614 m^2^; μ is the proportion of skin exposure, 18% [33]; η is the adhesion factor of skin to soil, 0.2 mg·cm^−2^ [33]; and $\gamma_{i}$ is the absorption coefficient of contaminant *i* in the skin, 1.00 × 10^−3^, 1.6 × 10^−2^, 1.00 × 10^−3^, 6.00 × 10^−4^, 1.00 × 10^−3^, 1.00 × 10^−3^, and 2.08 × 10^−5^ for Cr, Ni, Cu, Zn, As, Cd, and Pb, respectively [10].

#### 2.3.3. Respiratory Exposure

The concentration of trace metals entering the body through breathing can be calculated as follows:

(6) $$Q_{b,i}=\frac{Q_{i}\times \omega \times IR_{b}\times ED_{a}\times \theta_{i}\times \left(fsp_{od}\times EFOD_{a}+fsp_{id}\times EFID_{a}\right)}{BW\times LT}\times 10^{-6}$$

where $Q_{b,i}$ is the concentration of contaminant entering the body through breathing, in mg·kg^−1^·day^−1^; *ω* is the concentration of suspended particulates in the air, 0.15 mg·m^−3^ [33]; $IR_{b}$ is the breathing rate, 14.5 m^3^·day^−1^ [33]; and $\theta_{i}$ is the absorption coefficient of contaminant *i* in the lungs, 0.016, 0.3, 0.2, 0.01, and 0.15 for Ni, Cu, Zn, Cd, and Pb, respectively [10]. The absorption coefficients of Cr and As in the lungs has not been reported in the literature and is assumed to be 1.00; $fsp_{od}$ is the fraction of soil suspended particles outdoors, 0.8 [33]; $EFOD_{a}$ refers to the outdoor exposure frequency of adults, 87.5 day·year^−1^ [33]; $fsp_{id}$ is the fraction of soil suspended particles outdoors, 0.5 [33]; and $EFID_{a}$ is the outdoor exposure frequency of adults, 262.5 day·year^−1^ [33].

### 2.4. Human Health Risk Assessment

The seven trace metals in the study, Cr, Ni, Cu, Zn, As, Cd, and Pb, all exhibit non-carcinogenic risks, among which Cr, Ni, As, and Cd also exhibit carcinogenic risk.

#### 2.4.1. Potential Non-Carcinogenic Risk

Based on exposure assessment, the non-carcinogenic risk of trace metal contaminants in all exposure routes was calculated using a dose–response assessment and risk characterization. It can be calculated using following equations:

(7) $$HQ_{i}=\overset{3}{\underset{j=1}{\sum }}\frac{Q_{ij}}{RfD_{ij}}$$

(8) $$HI=\overset{7}{\underset{i=1}{\sum }}HQ_{i}$$

where $HQ_{i}$ represents the potential non-carcinogenic risk index for contaminant *i*; $CDI_{ij}$ is the daily average exposure of contaminant *i* through route *j*, in mg·kg^−1^·day^−1^; *j* refers to the three exposure pathways of the human body, oral exposure (abbreviated as *f*), skin exposure (abbreviated as *s*) and respiratory exposure (abbreviated as *b*); $RfD_{ij}$ is the reference dose of non-carcinogenic contaminant *i* through exposure route *j*, in mg·kg^−1^·day^−1^ (Table 3); and *HI* is the total non-carcinogenic risk index through all exposure routes in the list. Trace metals are expected to have adverse effects on humans when the value of *HI* is more than 1.0 [9].

#### 2.4.2. Potential Carcinogenic Risk

Based on the exposure assessment results from all exposure routes, the carcinogenic risk from soil pollution can be calculated as follows:

(9) $$CR_{i}=\overset{3}{\underset{j=1}{\sum }}Q_{ij}\times SF_{ij}$$

(10) $$TCR=\overset{4}{\underset{i=1}{\sum }}CR_{i}$$

where $CR_{i}$ is the potential carcinogenic risk index of carcinogenic contaminant *i*; $SF_{ij}$ is the carcinogenic risk slope coefficient of carcinogenic contaminant *i* through exposure route *j*, in kg·day·mg^−1^ (Table 3); and *TCR* is the total carcinogenic risk of contaminants through all exposure routes. The carcinogenic risk is negative when *CR_i_* or *TCR* ≤ 10^−6^; the maximum acceptable risk value is when *CR_i_* or *TCR* is 10^−4^.

### 2.5. Statistical Analysis

The normality of variances was checked by the Shapiro–Wilk test. Significant differences (*p* < 0.05) between organic fertilizers for different trace metals were assessed by ANOVA, and correlation analysis (Spearman’s r) was performed between the concentrations of trace metals in organic fertilizers. All statistical analyses above were performed with the SPSS 17.0 software (IBM, Armonk, NY, USA). Monte Carlo simulations were carried out to predict the time scale (in years) during which the trace metal content of farmland soil increase from background level to the maximum permissible limit as a result of using organic fertilizer. The simulations were performed with Crystal Ball 11.1software (Oracle Corporation, Redwood City, CA, USA). All figures were performed with the Microsoft Visio 2003 (Microsoft Corporation, Redmond, WA, USA), ArcGIS10.2 (Esri, Redlands, CA, USA), and Origin 2017 (OriginLab, Northampton, MA, US) software. All data were expressed on a dry weight (DW) basis.

## 3. Results and Discussion

### 3.1. Characteristics of Trace Metal Concentration in Organic Fertilizers

**Figure 2:**
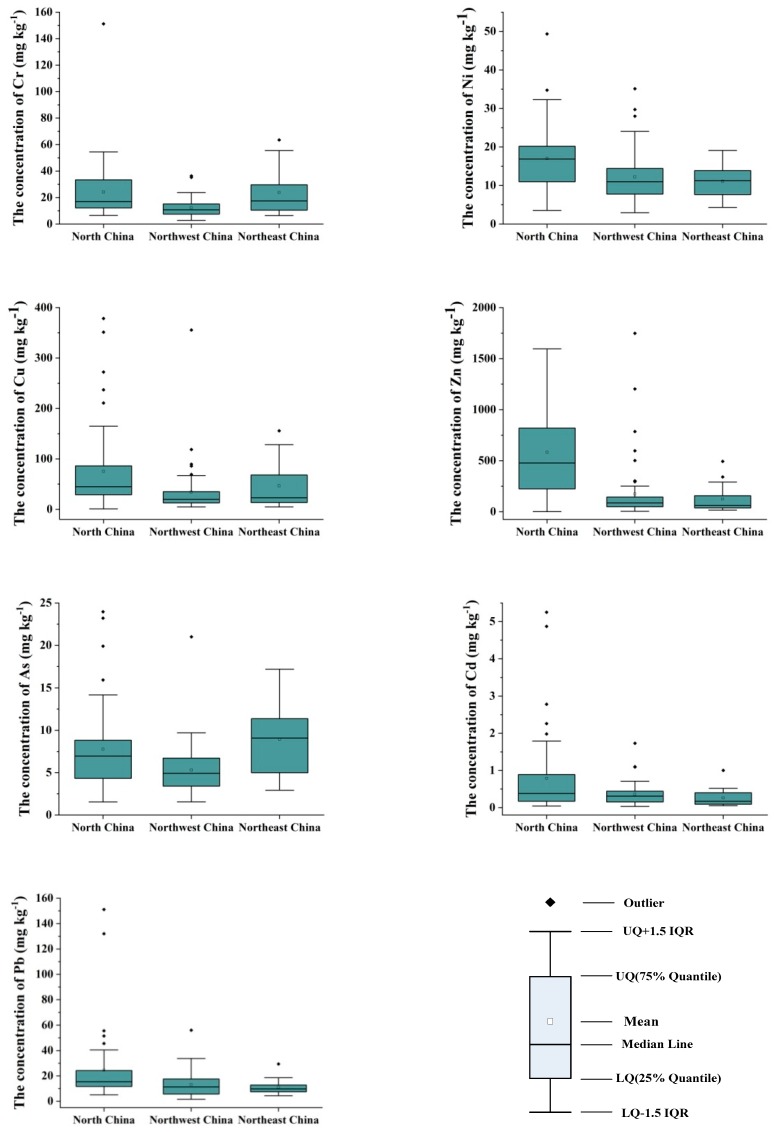
Trace metal concentrations of organic fertilizers in North China (*n* = 48), Northwest China (*n* = 55), and Northeast China (*n* = 14). Squares represent mean values, the band near the middle of each box represents the median, and the bottom and top of the boxes represent the lower quartile (LQ; 25th percentiles) and upper quartile (UQ; 75th percentiles), respectively. The vertical lines (whiskers) represent the 1.5 inter-quartile ranges (IQRs) of the lower and upper quartiles. Data outside the whiskers are outliers and are plotted as dots.

The measured concentration of trace elements in the organic fertilizer was widely variable according to both the element analyzed and the region (Table 4, Figure 2). Among the 48 samples collected in North China, the trace metal contents varied significantly (Cr: 6.53–151.15; Ni: 3.54–16.9; Cu: 0.76–378.32; Zn: 0.50–1595.42; As: 1.54–23.96; Cd: 0.04–5.25; and Pb: 5.08–151.09 mg·kg^−1^), with variability coefficients of 50.38–138.36%. Among the 55 samples taken from Northwest China, the trace metal contents also varied significantly (Cr: 2.74–36.34; Ni: 2.94–35.11; Cu: 4.55–355.52; Zn: 4.11–1748.01; As: 1.55–21.00; Cd: 0.03–1.73; and Pb: 1.60–55.98 mg·kg^−1^), with variability coefficients of 53.17–170.92%. Among the 14 samples from Northeast China, the trace metal contents were Cr: 6.43–63.46; Ni: 4.33–19.09; Cu: 4.69–155.68; Zn: 16.09–493.15; As: 2.93–17.19; Cd: 0.05–1.00; and Pb: 4.43–29.34 mg·kg^−1^, with variability coefficients of 41.39–117.19%.

It can be seen that the median contents of most trace metals in organic fertilizer in Northwest China were lower than those in other regions, due to the fact that its main raw material was cattle and sheep manure (Table 1). According to the research of Liu et al., the content of trace metals in manures followed the trend of pig manure > poultry manure > cattle manure > sheep manure [14] (Table 5). The Ni, Cu, Zn, Cd, and Pb contents of organic fertilizer in North China were the highest, and the Cr and As contents in Northeast China were the highest of the research areas, which was related to their raw materials (Table 1).

The trace metal contents of organic fertilizer in Northern China (0.03–1748.01 mg·kg^−1^) were lower than those in China (0.012–3692 mg·kg^−1^) and Southern China (Zhejiang Province: 0.1–5712 mg·kg^−1^; Jiangsu Province:*N*–11,378.9 mg kg^−1^). This might be due to the fact that the raw material used in Northern China was cattle, poultry, and sheep manure instead of pig manure. The concentration of trace metals in organic fertilizers in China is significantly higher than that reported in Switzerland (0–1021 mg·kg^−1^), Australia (<0.02–1439 mg·kg^−1^), and the UK (<0.10–780 mg·kg^−1^). While China is working to reduce the concentration of trace metals in organic fertilizer, according to Ding et al., the mean value of trace metal content tended to decrease with time in commercial composts from 2002–2013 [17].

### 3.2. Limit Standards and Exceeding Standards for Trace Metals in Organic Fertilizers

Trace metals have always been the focus in organic fertilizer standards. Establishing and implementing limit standards for trace metals in organic fertilizers could prevent agricultural pollution. Compliance with these standards ensures that fertilizers are safe for use; however, there is no guarantee that they will meet the specific need of end use [40]. Table 5 lists the maximum acceptable concentrations of Cr, Ni, Cu, Zn, As, Cd, and Pb in organic fertilizers in 24 regions of 20 countries. Some regions (Canada, Germany, Hong Kong, Taiwan, etc.) are set for different uses [41]. Different trace metals have higher threshold limits in agriculture/organic agriculture and lower thresholds for nonagricultural use. These standards have been established over many years of research, and different backgrounds and applications have led to large differences in standards among various countries. As seen in Table 6, the maximum acceptable concentrations of trace metals in the United States are significantly higher than those in other countries. Among these, the requirements in Mauritius and New Zealand are stringent. At present, organic fertilizer standards are not fixed, and some countries including China are exploring this field. At the end of 2017, Zhaocong Shang released the “fertilizer classification” on the official website of the Ministry of Industry and Information Technology of the People’s Republic of China [42]. The standard is a draft of the requirements, and divides all commercial fertilizers into farmland and ecological grades and sets different limits for trace metals. However, Cu and Zn are still not regulated by Chinese standards. In this survey, based on the limits of Cr, As, Cd, and Pb in the Chinese organic fertilizer standards and the regulations of Cu, Zn, and Ni in the German organic fertilizer standard class I (Table 6), the above-standard levels for Cu and Zn were the highest, followed by As and Pb; the rates for Cr, Ni, and Cd were the lowest. Although Cu and Zn are essential nutrients for agricultural soils, the introduction of excessive concentrations into the food chain may pose a threat to human health [43]. Therefore, monitoring and setting Cu and Zn limits for organic fertilizers is of great significance to prevent the accumulation of trace metals in soils, especially when the raw materials in organic fertilizers are livestock manure and sewage sludge [44,45].

Of all 117 samples, the Chinese Organic Fertilizer Standard limits were exceeded by 0.85% for Cr, 5.98% for As, 1.71% for Cd, and 4.27% for Pb. In Germany, the maximum acceptable concentrations were exceeded by 1.71% for Ni, 17.09% for Cu, and 35.04% for Zn. Excessive trace metals in organic fertilizers were not consistent in different regions. All trace metals in North China exceeded the standard. The standard limits for Cr, Ni, Cu, Zn, As, Cd, and Pb were exceeded by 2.08%, 2.08%, 29.17%, 68.75%, 8.33%, 4.17%, and 8.33%, respectively. In Northwest China, Cr and Cd did not exceed the limit. The contents of Ni, Cu, Zn, As, and Pb exceeded the standard by 1.82%, 7.27%, 10.91%, 1.82%, and 1.82%, respectively. In Northeast China, only Cu, Zn, and As exceeded the limit, by 14.29%, 14.29%, and 14.29%, respectively. Source control is the most effective way to prevent organic fertilizers from polluting the ecological environment. Trace metals in inorganic fertilizers mainly control the trace metal content in feed additives [46].

### 3.3. Correlation Analysis of Trace Metal Concentrations in Organic Fertilizer

**Figure 3:**
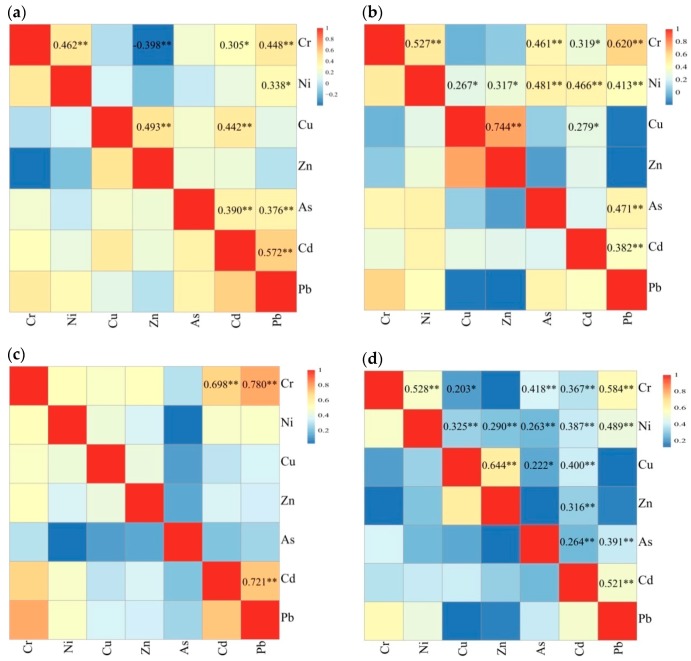
Spearman correlation analysis of trace metal concentrations in organic fertilizer. (**a**) organic fertilizers in North China (*n* = 48); (**b**) organic fertilizers in Northwest China (*n* = 55); (**c**) organic fertilizers in Northeast China (*n* = 14); and (**d**) organic fertilizers in northern China (*n* = 117). Degree of significance: * *p* < 0.05; ** *p* < 0.01.

All the data in this paper showed a skewed distribution (Table 4, Figure 2). Spearman correlation analysis was performed on seven trace metals. It can be seen from the correlation matrix (Figure 3) that there is only a low negative correlation (*p* < 0.01) between Cr and Zn in North China, so the negative correlation between trace metals in organic fertilizer was negligible, while the trace metal concentrations were positively correlated.

There were differences in the correlations between different trace metals in different regions. Cr and Pb were highly correlated in research area. Except for Northeast China, Cu and Zn, and Cr and Ni, were closely correlated. The correlation between Cd and Pb was high, except in Northwest China. Moderate correlations were observed between Cr and Pb, Cu and Cd (North China), Ni and As, As and Pb, Ni and Cd, Cr and As, Ni and Pb (Northwest China), and Cr and Cd (Northeast China).

A significant positive correlation between trace metals suggests a shared source. The relationship between Cu and Zn, Cr and Pb, Cr and Ni, and Cd and Pb was relatively close among the seven trace metals (Figure 3), and the content of these elements in organic fertilizer was high (Table 4, Figure 2). Source control is the most effective way to prevent organic fertilizers from polluting the ecological environment, and there is a significant positive correlation between the content of trace metals in organic manure and livestock manure corresponding to raw materials. The raw materials for the production of organic fertilizers in this paper were mainly livestock manure, while the trace metals in livestock manure were mainly from animal feed. The trace metals in organic fertilizers may be mostly derived from animal feeds high in Zn, Cu, Cr, and Pb [46]. Since Zn, Cu, Cr, and Pb were the four most abundant elements in the feeds, they represented a certain degree of environmental risk. It is important to limit their content. China’s current Feed Hygiene Standard (GB 13078-2017) does not regulate the highest levels of Zn and Cu, which may pose potential risks to the environment, even though these elements can improve the feed nutrients and pharmaceutical ingredients of livestock and poultry [43].

### 3.4. Accumulation Risk of Trace Metals in Soil from Organic Fertilizer

The long-term application of organic fertilizer containing trace metals can cause soil pollution. In order to estimate the time scale (in years) during which the trace metal content of farmland soil would increase from background level to the maximum permissible limit as a result of using organic fertilizer according to Soil Environmental Quality Standard of China (GB15618-2018), the data of annual application rate in the research area(Table 2) and the median concentrations of seven trace metals in the organic fertilizers were used (Table 5) [47].

According to Equations (2) and (3), the times needed to double the trace metal concentrations in soil from their background levels in North China, Northwest China, and Northeast China were 39, 46, and 46 years, respectively (Table 7). The simulation result was slightly overestimated, as the calculations did not consider the outputs of trace metals from the soil via leaching, runoff, and crop uptake, since these outputs were typically small compared with inputs [15,17,48]. Moreover, atmospheric deposition, which has a greater impact on trace metal accumulation in soil, was not counted. Nevertheless, this research shows the importance of setting the proper application amount and monitoring key trace metals in different regions. In North China, the average annual application rate could be higher, under the control of the content of Zn in organic fertilizer. In Northwest and Northeast China, the application should be reduced, as the time needed to double the trace metal concentrations in soil from their background levels was found to be short for Cd, As, and Zn (Table 7). Generally speaking, applying organic fertilizer, rather than chemical fertilizer, in agriculture is much better for soil quality and food safety. However, trace metal concentrations are usually high, and the soil fertility in Northern China has been decreasing annually [49,50]. In addition to monitoring the trace metal concentration and setting the proper application amount of organic fertilizer, other measures for fertility, such as no-tillage and fallow, can be explored.

In the process of simulating the accumulation of trace metals in the soil after the application of organic fertilizer, it was found that for the entire northern region of China, Zn, Cd, and As showed the highest risks, and Zn and Cd required the most attention as they were found to have the greatest impact on trace metal accumulation in soil during the 16 years of research of Wang et al. [47]. The highest-risk trace metals in different regions were different. In North China, Northwest China, and Northeast China, Zn, As, and Cd were the most important trace metals accumulated in trace metals. There was evidence that Zn, Cd, As, and Cu came from animal feed [51,52], since Zn and Cu were essential elements in commercial feeds to suppress bacteria and maximize feed utilization [25,53]. As it is used for disease control [51], concentrations of Cd were high in phosphorus-containing minerals used as animal feed ingredients, even though Cd is a nonessentialelement for animals [34,54]. Controlling the contents of Cu, Zn, As, and Cd in animal feed is the most effective way to reduce the risk of organic fertilizers causing the accumulation of trace metals in soil. In the current Feed Hygiene Standards, Organic Fertilizer Standards, and Bio-Organic Fertilizer Standards of China, there is no limit for the content of Zn, which poses risks to the environment. During the standard revision process, a limit value for Zn could be included by referring to values in other countries or regions (Table 6). Additionally, the limit for Cd in agricultural land in the Soil Environmental Quality Control and Control Standards for Soil Pollution Risk of Agricultural Land (Trial) (GB15618-2018), which is 0.6 mg·kg^−1^, differs largely from that in the China Organic Fertilizer Standard (NY525-2012), which is 3 mg·kg^−1^. Therefore, this could be adjusted appropriately during standard revision. The content of Cr, Ni, and Pb were higher in the soil environmental quality standard than those found in the background value of trace metals in northern China, and the limit could be appropriately lowered.

### 3.5. Risks of Trace Metals to Human Health through Soil

According to Equations (1) and (4)–(8), the non-carcinogenic risk of organic fertilizers was low, while the risk of Cr exposure on the skin was the highest (Table 8). Combined with Table 9, keeping the trace metal input constant and simulating the safe use of non-carcinogenic risk in the study area, HI = 1.00 is regarded as the maximum acceptable risk. Under the current fertilization level, North China, Northwest China, and Northeast China will experience adverse effects on human health after 403, 233, and 129 years, respectively, which are much longer than the time during which trace metal content of farmland soil will increase from background level to the maximum permissible limit. This might be due to the fact that the study only considered the effects of soil on human health but not the risk of human health in the food chain. More research on other exposure pathways in the study area is needed in the future [55,56,57].

**Figure 4:**
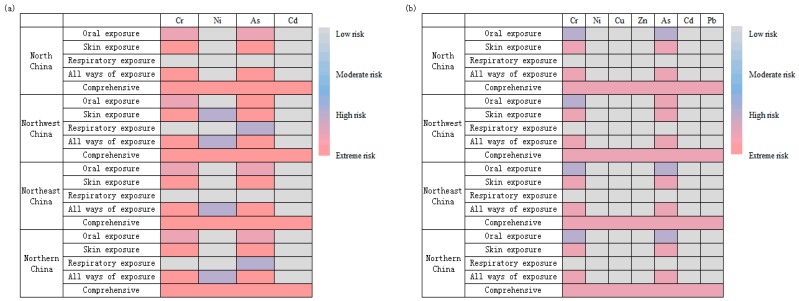
Risk level hotspot map of trace metals through oral, skin, and respiratory exposure pathways in North China, Northwest China, Northeast China, and northern China. (**a**) Carcinogenic risk level hotspot map of Cr, Ni, As, and Cd. (**b**) Human health risk level hotspot map of Cr, Ni, Cu, Zn, As, Cd, and Pb. Color description: orange-red represents extreme risk, purple-red represents high risk, purple-blue represents moderate risk, and gray represents low risk.

The carcinogenic risks for As and Cr were high for both oral and dermal absorption routes, while they were low or moderate for the inhalation of suspended particles (Figure 4). For all three exposure routes, As posed a higher risk to the human body than Cr (Table 7). Controlling the content of these two trace metals in organic fertilizer, especially As, can help prevent and control carcinogenic risks caused by the application of organic fertilizers in the soil. The results showed that arsenic in organic fertilizers was mainly As(V) (arsenate), followed by DMA (dimethylarsenite); organic arsenic in livestock manure was converted to inorganic arsenic during composting [58,59]. In addition to the prevention and control of As, the prevention of the conversion of organic arsenic into inorganic arsenic is an important research direction. Unfortunately, the risk assessments of As might be overestimated, with those conducted by the US-EPA having used faulty data in the dose–response estimation for cancer risk. There is an intense policy debate about the risk from As exposure, and final decisions may not be reached for years because of lawsuits that have been filed to challenge the change in cancer slope factor [60].

In general, human health (non-carcinogenic and carcinogenic) in northern China is considered to be at high risk (Figure 4, Table 10). Of the three exposure routes, skin exposure posed the highest risk; all trace metal elements produced a risk index that was at least half an order of magnitude higher than that for other exposure routes. Residents and agricultural workers in vegetable fields should minimize the chance of skin contact with the soil in production and life to reduce the risk to human health. Among the seven trace metals, the risks to human health from Cr and As were higher than those from other trace metals.

## 4. Conclusions

Reducing trace metal inputs in agricultural soils is an important strategy to protect farmland and ensure food safety. Estimation of the impact of the application of organic fertilizer on soil and human health is urgently needed to develop sound management practices and policies, which require information on the trace metals present in organic fertilizer. In this study, research was conducted in northern China to determine the metal contents in organic fertilizer.

A total of 117 organic fertilizer samples were collected, and concentrations of Cr (2.74–151.15 mg·kg^−1^), Ni (2.94–49.35 mg·kg^−1^), Cu (0.76–378.32 mg·kg^−1^), Zn (0.50–1748.01 mg·kg^−1^), As (1.54–23.96 mg·kg^−1^), Cd (2.74–151.15 mg·kg^−1^), and Pb (1.60–151.09 mg·kg^−1^) were measured. The contents of trace metals varied significantly, with variability coefficients of 54.23–146.32%. High correlations were observed between Cu and Zn, Cr and Pb, Cr and Ni, Cr and Cd, Ni and Pb, Cr and As, and Cu and Cd, which might have similar sources or cause co-pollution in the environment. Compared with the trace metal limit standards for organic fertilizers in 24 regions of the world, these trace metals exceeded the limits, showing different values. The Chinese organic fertilizer standards were exceeded for Cr (by 0.85%), As (by 5.98%), Cd (by 1.71%), and Pb (by 4.27%). The maximum acceptable concentrations of the German standard were exceeded by 1.71% for Ni, 17.09% for Cu, and 35.04% for Zn.

Monte Carlo simulations showed that after 39, 46, 46, and 53 years, the concentrations of Zn, Cd, and As will exceed the limits of the Soil Environmental Quality Standards of China in North China, Northwest China, Northeast China, and northern China, respectively. As and Cr pose high risks to human health, especially as carcinogenic risk factors with a skin exposure pathway. The simulation result was slightly overestimated; in future research, the outputs of organic fertilizer in the soil (leaching, runoff, and crop uptake) and the dose–response relationship of non-carcinogenic and carcinogenic risk factors should also be considered. It is important to control and monitor the additions of Cr, Zn, As, and Cd in organic fertilizer.

## Figures and Tables

**Table 1:** Proportion of manure used for making organic fertilizers in the research area.

Manure	North China (*n* = 48)		Northwest China (*n* = 55)		Northeast China (*n* = 14)	
	*n*	Proportion (%)	*n*	Proportion (%)	*n*	Proportion (%)
Cattle	2	4.17	18	32.73	5	35.72
Sheep	15	31.25	12	21.82	1	7.14
Poultry	12	25.0	3	5.45	4	28.57
Pig	0	0	6	10.91	0	0
Rabbit	2	4.17	0	0	0	0
Mixed	17	35.41	16	29.09	4	28.57

Note: *n* denotes the number of sampled organic fertilizers.

**Table 2:** Soil trace metal background value and organic fertilizer application rate.

Parameter	Unit	North China	Northwest China	Northeast China	References
Initial content of Cr in soil Q_0,Cr_	mg·kg^−1^	51.60	65.70	52.65	[27]
Initial content of Ni in soil Q_0,Ni_	mg·kg^−1^	25.75	32.55	22.10	[27]
Initial content of Cu in soil Q_0,Cu_	mg·kg^−1^	20.50	22.45	18.55	[27]
Initial content of Zn in soil Q_0,Zn_	mg·kg^−1^	67.30	69.25	75.55	[27]
Initial content of As in soil Q_0,As_	mg·kg^−1^	8.65	12.33	7.65	[27]
Initial content of Cd in soil Q_0,Cd_	mg·kg^−1^	0.091	0.115	0.093	[27]
Initial content of Pb in soil Q_0,Pb_	mg·kg^−1^	17.65	20.43	26.50	[27]
Soil bulk density (*ρ_b_*)	g·cm^−3^	1.35	1.18	1.41	[28]
Organic fertilizer application rate (IR_f_)	t·hm^−2^·year^−1^	33.92	80.30	115.89	[29,30,31,32]

Q_0,Cr_, Q_0,Ni_, Q_0,Cu_, Q_0,Zn_, Q_0,As_, Q_0,Cd_, Q_0,Pb_ refer to the initial content of Cr, Ni, Cu, Zn, As, Cd, Pb in the soil, respectively. *ρ*_b_ is the bulk density of soil, IR_f_ is the amount of organic fertilizer applied to the soil.

**Table 3:** Reference dose and slope coefficient of contaminants through different exposure routes (IRIS, US EPA).

Contaminants	SF	RfD_f_	RfD_s_	RfD_b_
	(kg·day·mg^−1^)	(mg·kg^−1^·day^−1^)	(mg·kg^−1^·day^−1^)	(mg·kg^−1^·day^−1^)
Cr	4.20 × 10^1^	3.00 × 10^−3^	2.9 × 10^−5^	6.00 × 10^−5^
Ni	8.40 × 10^−1^	2.00 × 10^−2^	2.00 × 10^−2^	5.40 × 10^−3^
Cu		4.00 × 10^−2^	4.00 × 10^−2^	1.20 × 10^−2^
Zn		3.00 × 10^−1^	3.00 × 10^−1^	6.00 × 10^−2^
As	1.51 × 10^1^	3.00 × 10^−4^	3.00 × 10^−4^	1.20 × 10^−3^
Cd	6.30	1.00 × 10^−3^	1.00 × 10^−3^	1.00 × 10^−5^
Pb		3.50 × 10^−3^	3.50 × 10^−3^	5.20 × 10^−4^

SF is the carcinogenic risk slope coefficient. RfD_f_, RfD_s_, RfD_b_arethe non-carcinogenic reference doses through the exposure routes of food, skin, and breathing, respectively.

**Table 4:** Trace metal concentrations in organic fertilizer in northern China (mg·kg^−1^).

		Cr	Ni	Cu	Zn	As	Cd	Pb
North China	Range	6.53–151.15	3.54–49.35	0.76–378.32	0.50–1595.42	1.54–23.96	0.04–5.25	5.08–151.09
	Mean	24.14	17.01	75.19	581.91	7.76	0.79	24.47
	Median	17.02	16.90	44.85	478.11	6.96	0.38	15.46
	SD	22.55	8.57	83.83	429.65	4.89	1.09	27.27
	C.V.(%)	93.43	50.38	111.50	73.83	63.02	138.36	111.46
	P_S-K_	0.000	0.003	0.000	0.006	0.000	0.000	0.000
Northwest China	Range	2.74–36.34	2.94–35.11	4.55–355.52	4.11–1748.01	1.55–21.00	0.03–1.73	1.60–55.98
	Mean	12.26	12.21	34.20	174.08	5.30	0.35	13.05
	Median	10.75	10.98	19.84	86.71	4.92	0.31	11.36
	SD	6.75	6.49	49.72	297.55	3.04	0.29	9.23
	C.V.(%)	55.03	53.17	145.39	170.92	57.35	82.75	70.70
	P_S-K_	0.000	0.000	0.000	0.000	0.000	0.000	0.000
Northeast China	Range	6.43–63.46	4.33–19.09	4.69–155.68	16.09–493.15	2.93–17.19	0.05–1.00	4.43–29.34
	Mean	23.75	11.09	46.53	124.53	8.93	0.26	10.99
	Median	17.47	11.23	23.02	60.86	9.08	0.17	9.73
	SD	17.89	4.59	46.83	145.93	4.50	0.26	6.49
	C.V.(%)	75.35	41.39	100.64	117.19	50.40	100.42	59.09
	P_S-K_	0.023	0.713	0.010	0.001	0.524	0.001	0.007
Northern China	Range	2.74–151.15	2.94–49.35	0.76–378.32	0.50–1748.01	1.54–23.96	0.03–5.25	1.60–151.09
	Mean	18.51	14.05	52.49	335.47	6.74	0.52	17.49
	Median	13.03	12.05	31.67	144.14	5.96	0.31	12.41
	SD	17.27	7.62	67.97	401.56	4.27	0.76	19.50
	C.V.(%)	93.28	54.23	129.50	119.70	63.37	146.32	111.50
	P_S-K_	0.000	0.000	0.000	0.000	0.000	0.000	0.000

SD: standard deviation, C.V.: Coefficient of Variance, P_S-K_: *p*-value of Shapiro–Wilk test.

**Table 5:** Trace metal concentrations in compost in published articles (based on median/mean value) (mg·kg^−1^).

Region	Type of Organic Fertilizer	*n*	Cr	Ni	Cu	Zn	As	Cd	Pb	References
Austria	Compost	NA	38.3	25.7	100	267	7.0	0.43	43.4	[34]
	Organic fertilizer: poultry dung	NA	10.7	8.5	66	314	NA	0.43	5.4	
	Organic fertilizer: pig dung	NA	7.8	8.9	62	399	NA	0.33	5.0	
UK ^a^	Straw-based farmyard dairy cattle manure	6	5.32	3.7	37.5	153	1.63	0.38	3.61	[35]
	Straw-based farmyard beef cattle manure	12	1.41	2.0	16.4	81	0.79	0.13	1.95	
	Straw-based farmyard pig manure	17	1.98	7.5	374	431	0.86	0.37	2.94	
Canada ^a^	Peat/manure compost	11	3.53 ± 0.51	3.8 ± 1.6	16.9 ± 7.3	142 ± 61	NA	0.26 ± 0.15	4.8 ± 2.2	[36]
	Straw/manure compost	14	4.5 ± 1.1	5.4 ± 1.8	25.1 ± 7.2	159 ± 35	NA	0.21 ± 0.05	1.7 ± 1.7	
Switzerland	Compost	81	20	16	52	147	NA	0.11	38	[12]
China	Animal manure composts	212	17.90	12.40	50.60	288.0	6.65	0.14	7.61	[13]
China	Commercial organic fertilizer	162	53.5	21	75.4	732.4	2.96	5.64	36.6	[14]
China	Commercial organic fertilizers	126	165.2 ± 583.2	NA	122.2 ± 166.8	260.6 ± 272.1	5.6 ± 4.0	1.2 ± 0.97	34.3 ± 112	[37]
North China	Livestock manure organic fertilizer	42	45.42	16.5	69.22	274.58	3.21	0.21	87.4	[38]
Jiangsu Province, China	Composite pig manure	80	8.0	NA	113.7	427.2	0.9	0.37	3.8	[25]
	Composite dairy manure	35	9.9	NA	45.5	186.3	1.0	0.32	8.4	
	Composite poultry manure	65	9.8	NA	56.7	391.2	1.5	0.42	4.5	
Zhejiang Province, China	Manure-based fertilizers	219	21.2	NA	160	465	7.9	0.6	8.1	[39]

^a^ mean value; NA, not available in the article.

**Table 6:** Maximum trace metal concentration acceptable for compost in different countries (mg·kg^−1^).

Region	Cr	Ni	Cu	Zn	As	Cd	Pb	Standard File
Africa								
Mauritius	50	50	-	300	10	3	100	MS 164 (2010)
America								
United States	1200	420	1500	2800	41	39	300	USCC-2001
United States, Washington	-	210	750	1400	20	10	150	WSDA, 2009
Canada A	210	62	400	700	13	3	150	CCME-2005
Canada B	1060	180	757	1850	75	20	500	CAN/BNQ/CCME/AAFC
Europe								
EU organic agriculture	70	25	70	200	-	0.7	45	Biowaste Directory
EU ecological standards	100	50	100	300	10	1	100	Biowaste Directory
EU soil amendment	100	50	100	300	-	1	100	Biowaste Directory
United Kingdom	100	50	200	400	-	1.5	200	BSI-PAS 100-2011
France	120	60	300	600	-	3	180	NF U44-051
Netherlands	50	20	90	290	-	1	100	Amended National Fertilizer Act from 2008
Italy	-	50	150	500	-	1.5	140	Law on Fertilizer L 748/84
Belgium	100	50	150	400	20	2	150	Royal Decree
Denmark	-	30	1000	4000	25	0.8	-	Statutory Oder Nr.1650
Spain A	70	25	70	200	-	0.7	45	Spain RD 506-2013
Spain B	250	90	300	500	-	2	150	Spain RD 506-2013
Spain C	300	100	400	1000	-	3	200	Spain RD 506-2013
Germany I	70	35	70	300	-	1	100	Biowaste Ordinance
Germany Ⅱ	100	50	100	400	-	1.5	150	
Switzerland	100	30	100	400	-	1	120	Swiss Federal Council-2013
Oceania								
New Zealand compost	50	10	25	75	5	0.7	65	SDU-1991
New Zealand clean compost	50	20	60	200	15	1	100	SDU-1991
Aureli A^+^	70	25	70	200	-	0.7	45	SWD 64/F1-EN,2016
Aureli A	70	60	150	500	-	1	120	SWD 64/F1-EN,2016
Australia	400	60	200	250	20	3	200	Compost Ordinance/AS 4454-2012
Asia								
Malaysia	200	150	-	-	-	5	300	MS 1517-2012
India	50	50	-	1000	10	3	150	World Bank, 1997
Indonesia	50	50	-	300	10	3	100	Ministry of Environment and Forests
Japan	500	300	-	-	50	5	100	Fertilizer Management Act
Korea	300	50	500	900	5	5	150	Fertilizer Management Act
Hong Kong organic agriculture	100	50	300	600	10	1	100	Hong Kong ORC, 2005
Hong Kong general agricultural use	210	62	700	1300	13	3	150	Hong Kong ORC, 2005
Hong Kong nonagricultural use	1200	420	1500	2800	41	19	300	Hong Kong ORC, 2005
Taiwan livestock and poultry composting	150	25	100	500	25	2	150	Fertilizer Management Act
Taiwan general compost	150	25	100	250	25	2	150	Fertilizer Management Act
Taiwan miscellaneous compost	150	25	100	250	25	2	150	Fertilizer Management Act
Mainland China	300	-	-	-	30	3	100	NY525-2012

**Table 7:** Input of trace metals and time needed to double the trace metal concentrations of soil from its background levels in the research area.

Area		Cr	Ni	Cu	Zn	As	Cd	Pb
North China	Amount of application (mg·kg^−1^)	0.21	0.21	0.56	6.01	0.087	0.0048	0.19
	Time (year)	928	774	141	39	187	107	784
Northwest China	Amount of application (mg·kg^−1^)	0.37	0.37	0.68	2.95	0.17	0.011	0.39
	Time (year)	504	421	115	78	76	46	387
Northeast China	Amount of application (mg·kg^−1^)	0.72	0.46	0.95	2.50	0.37	0.0070	0.40
	Time (year)	275	364	86	90	46	73	359
Northern China	Amount of application (mg·kg^−1^)	0.39	0.36	0.96	4.36	0.18	0.0094	0.38
	Time (year)	484	443	82	53	82	53	396

**Table 8:** Non-carcinogenic risk indices in the study area.

	Exposure Route	Cr	Ni	Cu	Zn	As	Cd	Pb
North China	Oral exposure	1.81 × 10^−4^	8.70 × 10^−6^	2.21 × 10^−4^	1.04 × 10^−4^	1.22 × 10^−2^	2.01 × 10^−6^	2.14 × 10^−3^
	Skin exposure	1.09 × 10^−1^	5.05 × 10^−5^	1.28 × 10^−3^	5.95 × 10^−4^	7.09 × 10^−2^	1.17 × 10^−5^	1.24 × 10^−2^
	Respiratory exposure	1.58 × 10^−4^	5.64 × 10^−7^	1.29 × 10^−5^	8.96 × 10^−6^	5.34 × 10^−5^	3.51 × 10^−6^	2.52 × 10^−4^
	All ways of exposure	1.09 × 10^−1^	5.98 × 10^−5^	1.51 × 10^−3^	7.06 × 10^−4^	8.31 × 10^−2^	1.72 × 10^−5^	1.48 × 10^−2^
	Comprehensive	0.21						
Northwest China	Oral exposure	2.31 × 10^−4^	1.10 × 10^−5^	2.42 × 10^−4^	1.01 × 10^−4^	1.74 × 10^−2^	2.63 × 10^−6^	2.50 × 10^−3^
	Skin exposure	1.39 × 10^−1^	6.41 × 10^−5^	1.41 × 10^−3^	5.86 × 10^−4^	1.01 × 10^−1^	1.53 × 10^−5^	1.45 × 10^−2^
	Respiratory exposure	2.02 × 10^−4^	7.15 × 10^−7^	1.41 × 10^−5^	8.82 × 10^−6^	7.63 × 10^−5^	4.60 × 10^−6^	2.93 × 10^−4^
	All ways of exposure	1.39 × 10^−1^	7.58 × 10^−5^	1.67 × 10^−3^	6.95 × 10^−4^	1.19 × 10^−1^	2.25 × 10^−5^	1.73 × 10^−2^
	Comprehensive	0.28						
Northeast China	Oral exposure	1.86 × 10^−4^	7.56 × 10^−6^	2.04 × 10^−4^	1.09 × 10^−4^	1.12 × 10^−2^	2.09 × 10^−6^	3.22 × 10^−3^
	Skin exposure	1.12 × 10^−1^	4.39 × 10^−5^	1.19 × 10^−3^	6.33 × 10^−4^	6.51 × 10^−2^	1.22 × 10^−5^	1.87 × 10^−2^
	Respiratory exposure	1.63 × 10^−4^	4.90 × 10^−7^	1.19 × 10^−5^	9.54 × 10^−6^	4.90 × 10^−5^	3.67 × 10^−6^	3.79 × 10^−4^
	All ways of exposure	1.12 × 10^−1^	5.20 × 10^−5^	1.40 × 10^−3^	7.51 × 10^−4^	7.63 × 10^−2^	1.79 × 10^−5^	2.23 × 10^−2^
	Comprehensive	0.21						
Northern China	Oral exposure	2.07 × 10^−4^	9.58 × 10^−6^	2.30 × 10^−4^	1.04 × 10^−4^	1.45 × 10^−2^	2.35 × 10^−6^	2.59 × 10^−3^
	Skin exposure	1.24 × 10^−1^	5.57 × 10^−5^	1.34 × 10^−3^	6.06 × 10^−4^	8.45 × 10^−2^	1.37 × 10^−5^	1.50 × 10^−2^
	Respiratory exposure	1.81 × 10^−4^	6.21 × 10^−7^	1.34 × 10^−5^	9.13 × 10^−6^	6.37 × 10^−5^	4.12 × 10^−6^	3.05 × 10^−4^
	All ways of exposure	1.25 × 10^−1^	6.59 × 10^−5^	1.58 × 10^−3^	7.19 × 10^−4^	9.91 × 10^−2^	2.01 × 10^−5^	1.79 × 10^−2^
	Comprehensive	0.24						

**Table 9:** Carcinogenic risk indices and risk rating in the study area.

	Exposure Route	Cr	Ni	As	Cd
North China	Oral exposure	2.28 × 10^−5^	1.46 × 10^−7^	5.52 × 10^−5^	1.26 × 10^−8^
	Skin exposure	1.33 × 10^−4^	8.49 × 10^−7^	3.21 × 10^−4^	7.34 × 10^−8^
	Respiratory exposure	3.99 × 10^−7^	2.56 × 10^−9^	9.67 × 10^−7^	2.21 × 10^−10^
	All ways of exposure	1.56 × 10^−4^	9.98 × 10^−7^	3.77 × 10^−4^	8.62 × 10^−8^
	Comprehensive	5.34 × 10^−4^			
Northwest China	Oral exposure	2.90 × 10^−5^	1.85 × 10^−7^	7.90 × 10^−4^	1.66 × 10^−8^
	Skin exposure	1.69 × 10^−4^	1.08 × 10^−6^	4.59 × 10^−4^	9.62 × 10^−8^
	Respiratory exposure	5.09 × 10^−7^	3.24 × 10^−9^	1.38 × 10^−6^	2.90 × 10^−10^
	All ways of exposure	1.98 × 10^−4^	1.27 × 10^−6^	5.40 × 10^−4^	1.13 × 10^−7^
	Comprehensive	7.39 × 10^−4^			
Northeast China	Oral exposure	2.35 × 10^−5^	1.27 × 10^−7^	5.07 × 10^−5^	1.32 × 10^−8^
	Skin exposure	1.36 × 10^−4^	7.38 × 10^−7^	2.95 × 10^−4^	7.66 × 10^−8^
	Respiratory exposure	4.11 × 10^−7^	2.22 × 10^−9^	8.88 × 10^−7^	2.31 × 10^−10^
	All ways of exposure	1.60 × 10^−4^	8.67 × 10^−7^	3.46 × 10^−4^	9.00 × 10^−8^
	Comprehensive	5.08 × 10^−4^			
Northern China	Oral exposure	2.61 × 10^−5^	1.61 × 10^−7^	6.59 × 10^−5^	1.48 × 10^−8^
	Skin exposure	1.51 × 10^−4^	9.35 × 10^−7^	3.83 × 10^−4^	8.61 × 10^−8^
	Respiratory exposure	4.56 × 10^−7^	2.82 × 10^−9^	1.15 × 10^−6^	2.60 × 10^−10^
	All ways of exposure	1.78 × 10^−4^	1.10 × 10^−6^	4.50 × 10^−4^	1.01 × 10^−7^
	Comprehensive	6.29 × 10^−4^			

**Table 10:** Potential human health risk grade.

Potential Human Health Risk Grade			Potential Non-carcinogenic Risk			
			Low Risk	Moderate Risk	High Risk	Extreme Risk
			≤0.25]	(0.25–0.50]	(0.50–0.75]	(0.75–1.00]
Potential cancer risk	Low risk	≤10^−6^	Low risk	Low risk	Moderate risk	High risk
	Moderate risk	(10^−6^–10^−5^]	Low risk	Moderate risk	Moderate risk	High risk
	High risk	(10^−5^–10^−4^]	Moderate risk	Moderate risk	High risk	Extreme risk
	Extreme risk	≥10^−4^	High risk	High risk	Extreme risk	Extreme risk
